# Supplementary material for: A unique bacterial tactic to circumvent the cell death crosstalk induced by blockade of caspase‐8
Source: EMBO J. 2020 Jul 13;39(17):e104469. doi: 10.15252/embj.2020104469 (PMC7459423; doi:10.15252/embj.2020104469)
Supplement: Supplementary file 1 — Appendix [file EMBJ-39-e104469-s001.pdf]

# A unique bacterial tactic to circumvent the cell-death crosstalk induced by blockade of caspase-8

**Hiroshi Ashida, Chihiro Sasakawa, and Toshihiko Suzuki**

**Appendix**

Appendix Figure S1

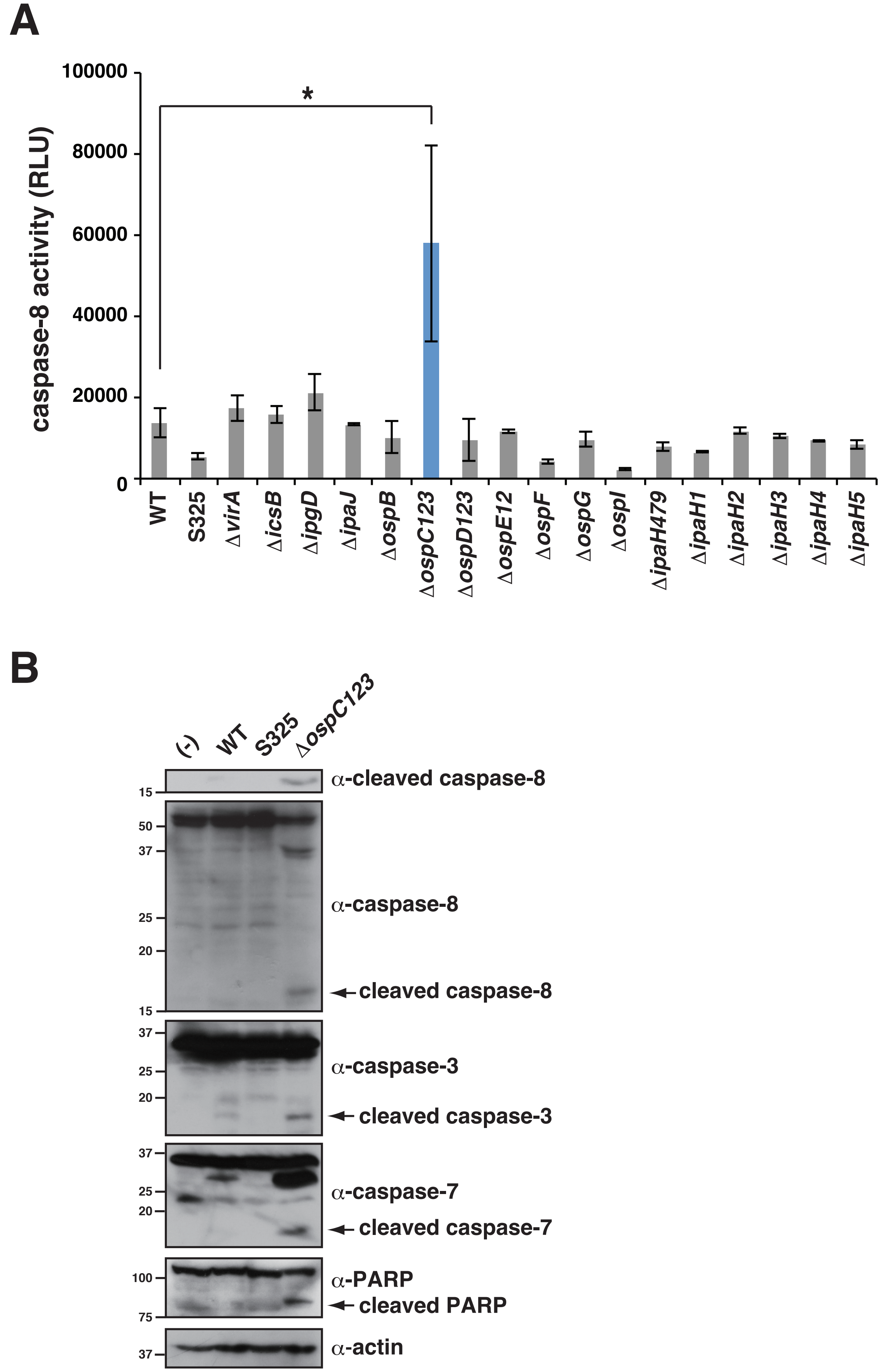


**Appendix Figure S1. *Shigella* OspC effector prevents caspase-8 activation**

(A) HT29 cells were infected with the indicated *Shigella* strains and incubated for 8 h. Cells were harvested and subjected to measurement of caspase-8 activation. Caspase-8 activity is reported as relative light units (RLU) of infected samples, minus the value in uninfected samples. *$P$<0.05 (one-way ANOVA).

(B) HT29 cells were infected with *Shigella* WT, S325, or *ΔospC123* strains, and then incubated for 8 h. Cell lysates were subjected to immunoblotting. Graph shows the mean ± SD, and data are pooled from three independent experiments performed in triplicates (A). Data are representatives of three independent experiments (B). Molecular weights in immunoblots are in kDa.

**Appendix Fig. S1**
