# Supplementary material for: A unique bacterial tactic to circumvent the cell death crosstalk induced by blockade of caspase‐8
Source: EMBO J. 2020 Jul 13;39(17):e104469. doi: 10.15252/embj.2020104469 (PMC7459423; doi:10.15252/embj.2020104469)
Supplement: Supplementary file 2 — Expanded View Figures PDF [file EMBJ-39-e104469-s002.pdf]

Expanded View Figures

Figure EV1. ***Shigella* Δ*ospD3* induces caspase-independent necrotic cell death.**

A HT29 cells were infected with the indicated *Shigella* strains in the presence or absence of caspase inhibitor (Z-VAD-fmk, 10 μM) and incubated for 8 h. Aliquots of cellular supernatants were subjected to cytotoxicity assays. n.s., not significant; *$P < 0.05$ (unpaired two-tailed Student's *t*-test).

B HT29 cells were infected with the indicated *Shigella* strains. After 8 h of incubation, infected cells were harvested and subjected to immunoblotting.

C HT29 cells were infected with *Shigella* WT, Δ*ospD3*, or Δ*ospC3* strains. Aliquot of cellular supernatants obtained at the indicated time points were subjected to cytotoxicity assay. n.s., not significant; *$P < 0.05$ (unpaired two-tailed Student's *t*-test).

D HT29 cells were infected with the indicated *Shigella* strains or stimulated with staurosporine, and then incubated for 8 h. Cells were harvested and subjected to measurement of caspase activity. n.s., not significant (one-way ANOVA).

Data information: Graphs in (A), (C), and (D) show mean ± SD, and data are pooled from three independent experiments performed in triplicates. Images in (B) are representative of three independent experiments. Molecular weights in immunoblots are in kDa.
Source data are available online for this figure.

© 2020 The Authors

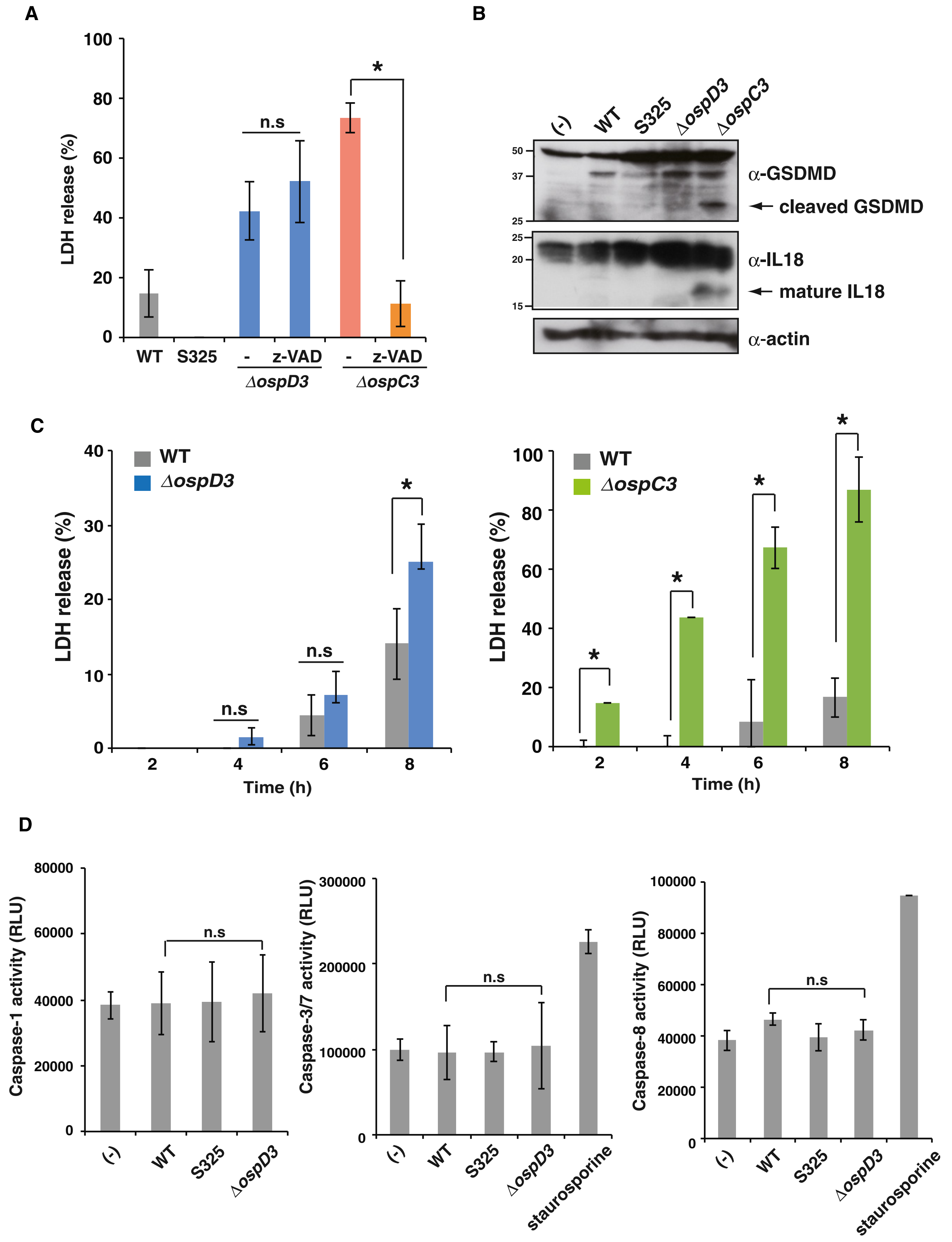

Figure EV1.

 © 2020 The Authors

Figure EV2. *Shigella* OspD3 specifically inhibits necroptosis.

A HT29, HeLa, HCT116, and HaCaT cells were infected with *Shigella* WT, S325, or *ospD3*, and then incubated at 37°C for 8 h. Aliquots of cellular supernatants were subjected to cytotoxicity assays. n.s., not significant; *$P < 0.05$ (unpaired two-tailed Student's *t*-test).

B Infected cells were harvested and subjected to immunoblotting.

C Cell lysates of each cell line were subjected to immunoblotting.

D, E HeLa cells stably expressing GFP or RIPK3 were infected with *Shigella* WT or Δ*ospD3* and then incubated for 8 h. Cell lysates and aliquots of cellular supernatants were subjected to immunoblotting (D) and cytotoxicity assay (E), respectively. *$P < 0.05$; n.s., not significant (unpaired two-tailed Student's *t*-test).

Data information: Graphs in (A) and (E) show mean ± SD, and data are pooled from three independent experiments performed in triplicates. Images in (B)–(D) are representative of three independent experiments. Molecular weights in immunoblots are in kDa.
Source data are available online for this figure.

© 2020 The Authors

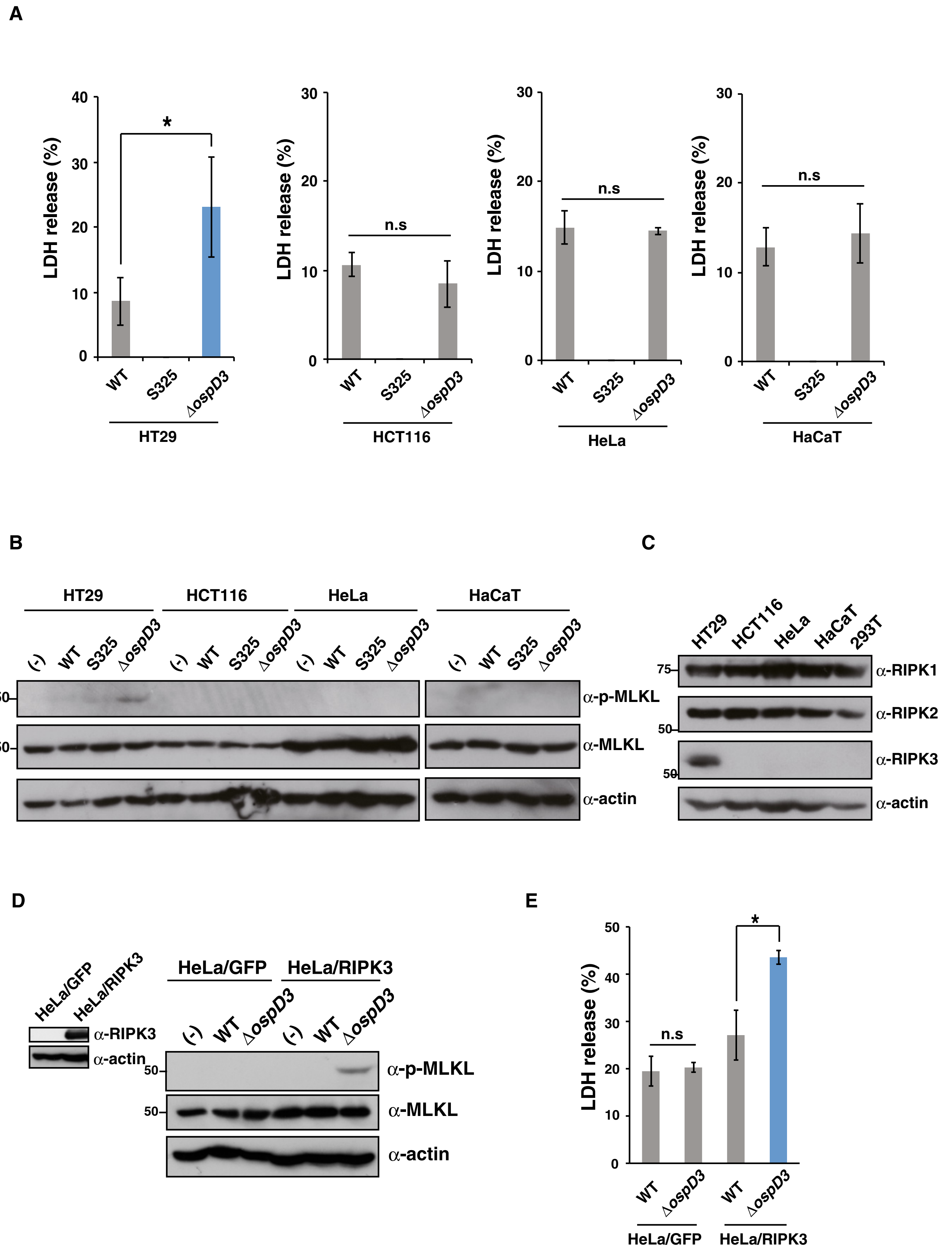

Figure EV2.

 © 2020 The Authors

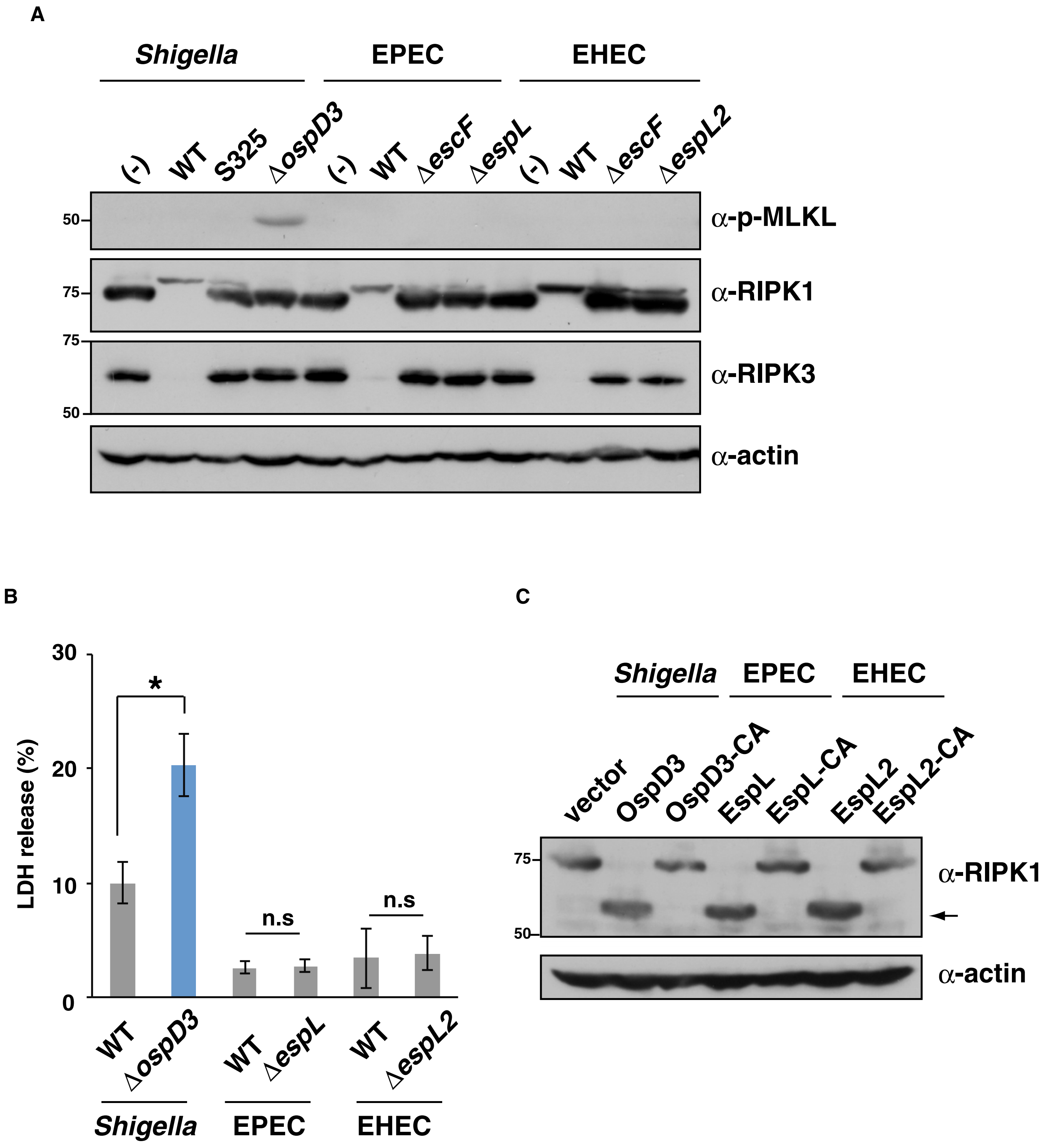
A
Shigella
EPEC
EHEC
(-)
WT
S325
ΔospD3
(-)
WT
ΔescF
ΔespL
(-)
WT
ΔescF
ΔespL2
50
75
75
50
α-p-MLKL
α-RIPK1
α-RIPK3
α-actin
B
LDH release (%)
30
20
10
0
*
n.s
n.s
WT
ΔospD3
WT
ΔespL
WT
ΔespL2
Shigella
EPEC
EHEC
C
Shigella
EPEC
EHEC
vector
OspD3
OspD3-CA
EspL
EspL-CA
EspL2
EspL2-CA
75
50
α-RIPK1
α-actin

Figure EV3.

Figure EV3. OspD3 homolog effector of EPEC and EHEC cleaves RIPK1 and RIPK3 via its C-H-D motif.

A HT29 cells were infected with the indicated *Shigella*, EPEC, or EHEC strains. After 8 h of incubation, infected cells were harvested and subjected to immunoblotting.
B HT29 cells were infected with the indicated *Shigella*, EPEC, or EHEC strains for 8 h. Aliquots of cellular supernatants were subjected to cytotoxicity assays. $^*P < 0.05$; n.s., not significant (unpaired two-tailed Student's *t*-test).
C 293T cells were transfected with a series of plasmids expressing point mutants of *ospD* homologs (the cysteine residue at position 64 of *Shigella ospD3* was replaced by alanine). After 24 h, the cells were harvested and subjected to immunoblotting. Arrows indicate cleaved RIPK1.

Data information: Graph in (B) shows mean $\pm$ SD, and data are pooled from three independent experiments performed in triplicates. Images in (A) and (C) are representative of three independent experiments. Molecular weights in immunoblots are in kDa.
Source data are available online for this figure.

Figure EV4. OspD3 cleaves the RHIM domain of RIPK1 and RIPK3.

A 293T cells were transfected with a series of RIPK1 truncations, along with plasmids expressing OspD3 or OspD3-CS (protease activity-deficient mutant, in which the cysteine residue at position 64 was replaced by serine). After 24 h, cells were harvested and subjected to immunoblotting. Asterisks indicate the non-cleaved form of truncated RIPK1.
B Sequence alignment of the RIPK1 and RIPK3 (top). 293T cells were transfected with a series of plasmids expressing the RIPK1 RHIM domain mutant (4A and 3A indicate alanine replacement of four and three amino acids, respectively, within the indicated regions of RIPK1) along with OspD3 or OspD3-CS. After 24 h, cells were harvested and subjected to immunoblotting (bottom).
C 293T cells were transfected with plasmids expressing RIPK1, RIPK1 RHIM mutant, RIPK3, or RIPK3 RHIM mutant along with empty vector, OspD3, or OspD3-CS. After 24 h, cells were harvested and subjected to immunoblotting.
D HeLa cells stably expressing RIPK3 or RIPK3-4A were infected with *Shigella* WT or Δ*ospD3*, and incubated for 8 h. Cell lysates were subjected to immunoblotting.

Data information: All data are representatives of three independent experiments. Molecular weights in immunoblots are in kDa.
Source data are available online for this figure.

 © 2020 The Authors

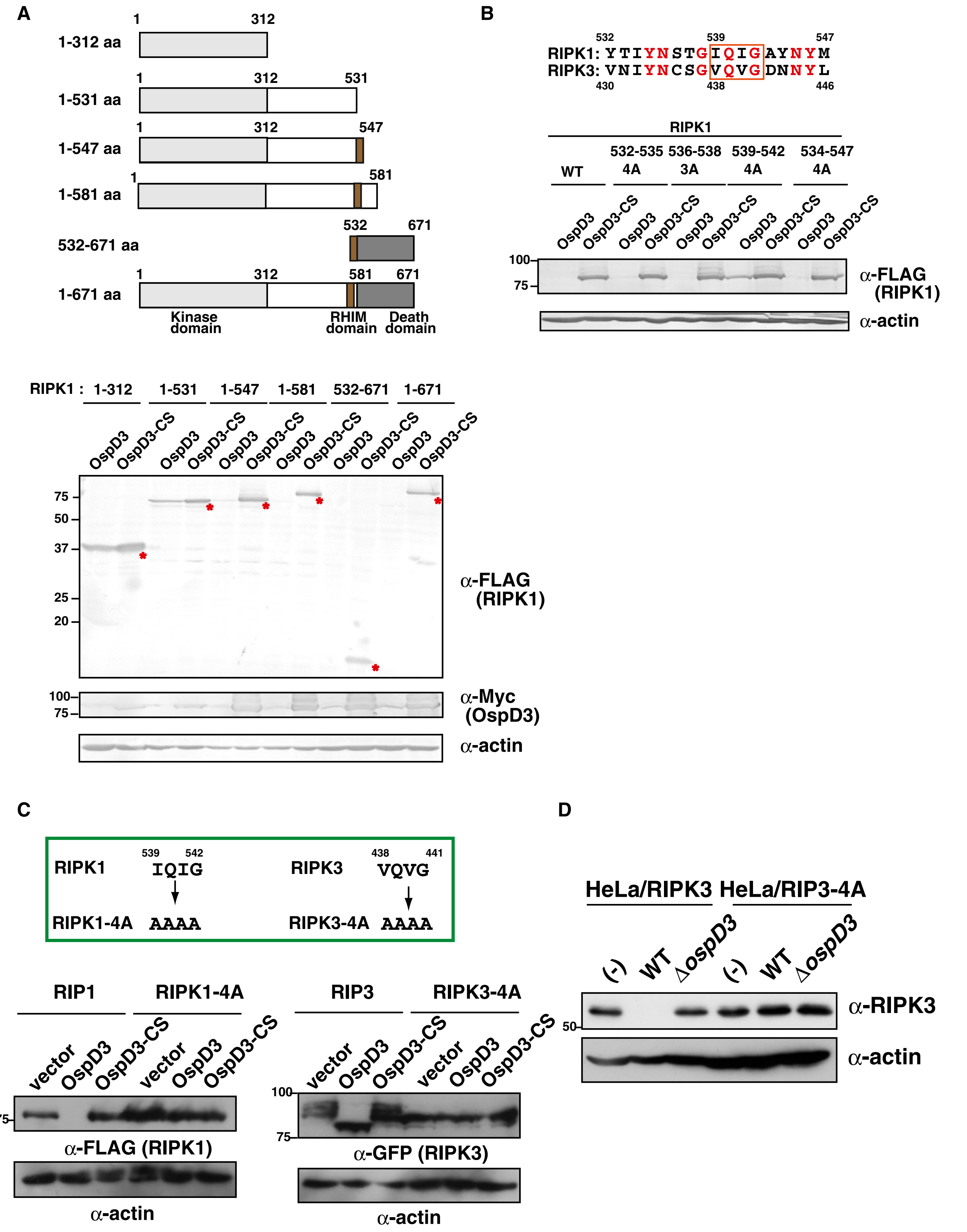
A
1-312 aa
1-531 aa
1-547 aa
1-581 aa
532-671 aa
1-671 aa
Kinase domain
RHIM domain
Death domain
B
RIPK1: YTIYNSTGIQIGAYNYM
RIPK3: VNIYNCSGVQVGDNNYL
RIPK1
WT
532-535 4A
536-538 3A
539-542 4A
534-547 4A
OspD3
OspD3-CS
α-FLAG (RIPK1)
α-actin
RIPK1 : 1-312 1-531 1-547 1-581 532-671 1-671
α-Myc (OspD3)
C
RIPK1 IQIG
RIPK1-4A AAAA
RIPK3 VQVG
RIPK3-4A AAAA
RIP1
RIPK1-4A
RIP3
RIPK3-4A
vector
α-GFP (RIPK3)
D
HeLa/RIPK3
HeLa/RIP3-4A
(-)
WT
ΔospD3
α-RIPK3

Figure EV4.

Figure EV5. *Shigella* OspC1-mediated caspase-8 inhibition triggers necroptosis.

A HT29 cells were infected with the indicated *Shigella* strains and incubated for 8 h. Cell lysates were subjected to immunoblotting.

B HT29 cells were infected with *Shigella* WT, Δ*ospD3*, or Δ*ospC1* strains. Cell lysates obtained at the indicated time points were subjected to immunoblotting.

C HT29 cells were infected with *Shigella* WT or Δ*ospC3* strains. Cell lysates obtained at the indicated time points were subjected to immunoblotting.

D HT29 cells treated with control or caspase-8 siRNAs were infected with the indicated *Shigella* strains and incubated for 8 h. Aliquots of cellular supernatants were subjected to cytotoxicity assay. *$P < 0.05$; n.s., not significant (unpaired two-tailed Student's *t*-test).

Data information: Graph in (D) shows mean ± SD, and data are pooled from three independent experiments performed in triplicates. Images in (A)–(C) are representative of three independent experiments. Molecular weights in immunoblots are in kDa.
Source data are available online for this figure.

 © 2020 The Authors

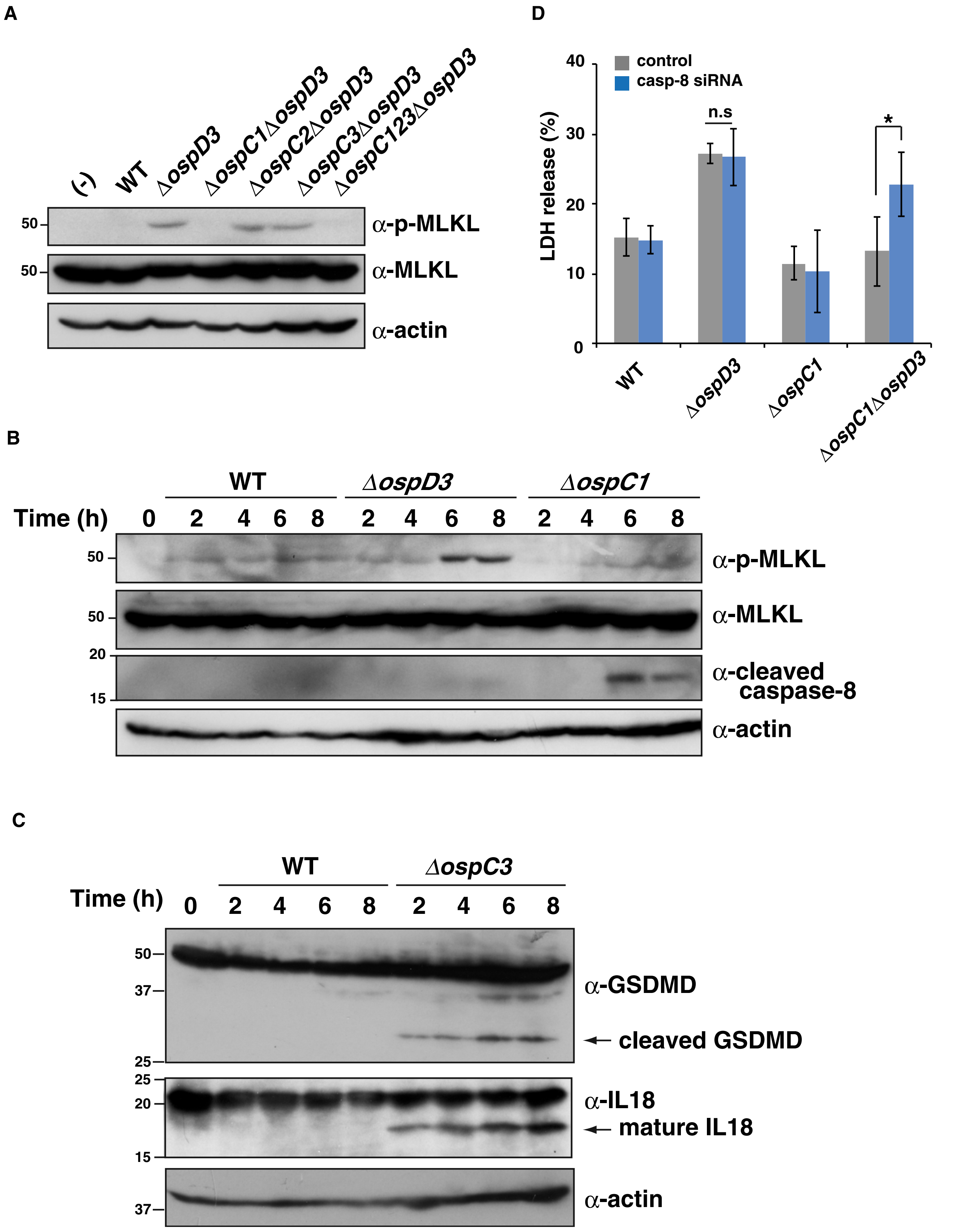
A
(-)
WT
ΔospD3
ΔospC1ΔospD3
ΔospC2ΔospD3
ΔospC3ΔospD3
ΔospC123ΔospD3
50
α-p-MLKL
50
α-MLKL
α-actin
D
control
casp-8 siRNA
LDH release (%)
40
30
20
10
0
n.s
*
WT
ΔospD3
ΔospC1
ΔospC1ΔospD3
B
WT
ΔospD3
ΔospC1
Time (h)
0 2 4 6 8 2 4 6 8 2 4 6 8
50
α-p-MLKL
50
α-MLKL
20
15
α-cleaved caspase-8
α-actin
C
WT
ΔospC3
Time (h)
0 2 4 6 8 2 4 6 8
50
37
25
α-GSDMD
cleaved GSDMD
25
20
15
α-IL18
mature IL18
37
α-actin

Figure EV5.
