# Supplementary material for: A unique bacterial tactic to circumvent the cell death crosstalk induced by blockade of caspase‐8
Source: EMBO J. 2020 Jul 13;39(17):e104469. doi: 10.15252/embj.2020104469 (PMC7459423; doi:10.15252/embj.2020104469)
Supplement: Supplementary file 3 — Source Data for Expanded View [file EMBJ-39-e104469-s007.zip › Source data for Fig. EV4.pdf]

Fig. EV4A

α-FLAG

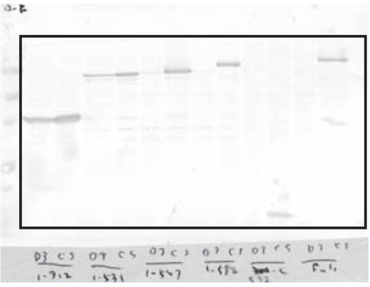

α-Myc

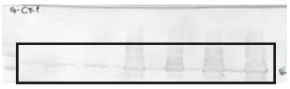

α-actin

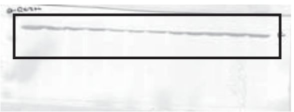

Fig. EV4B

α-FLAG

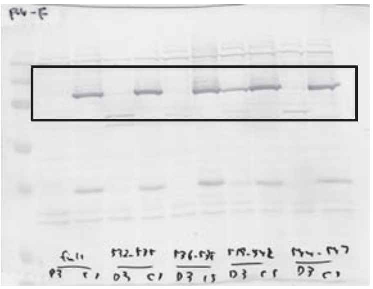

α-actin

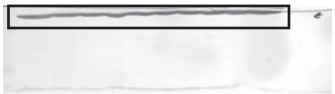

Fig. EV4C

α-FLAG

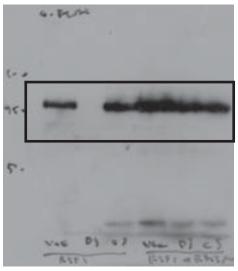

α-actin

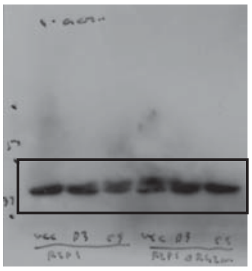

α-GFP

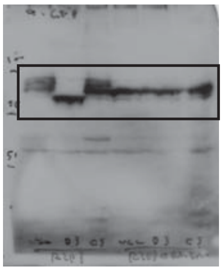

α-actin

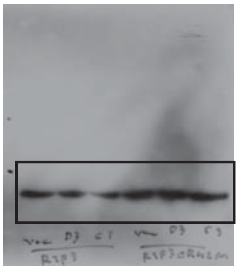

Fig. EV4D

α-RIPK3

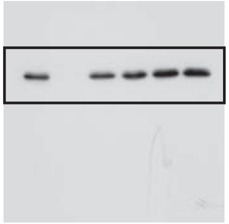

α-actin

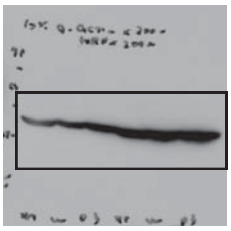

Source data for Fig. EV4
