# Supplementary figures and images for: A unique bacterial tactic to circumvent the cell death crosstalk induced by blockade of caspase‐8
Source: EMBO J. 2020 Jul 13;39(17):e104469. doi: 10.15252/embj.2020104469 (PMC7459423; doi:10.15252/embj.2020104469)

Appendix Fig. S1B

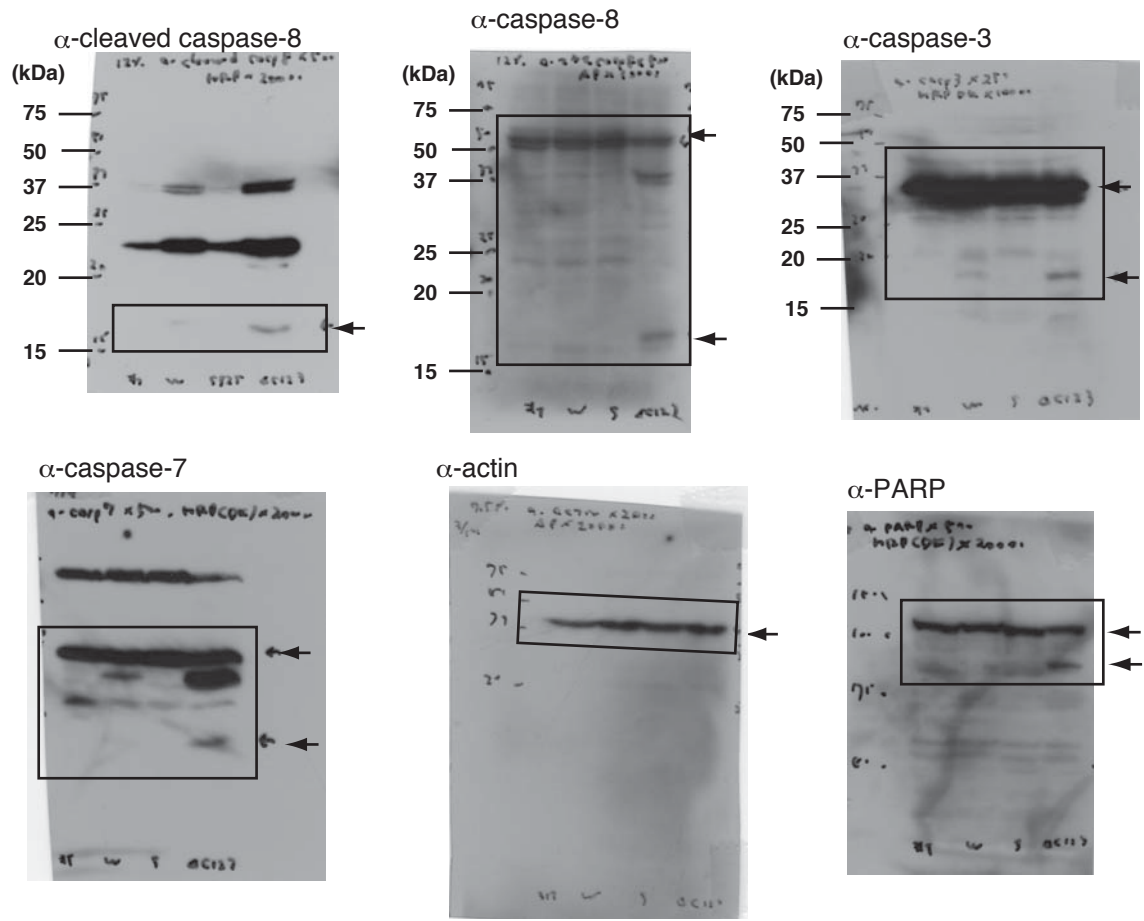

Source data for Appendix Fig. S1

Supplement: Supplementary file 3 — Source Data for Expanded View [file EMBJ-39-e104469-s007.zip › Source data for Appendix Fig. S1.pdf]

Fig. EV1B

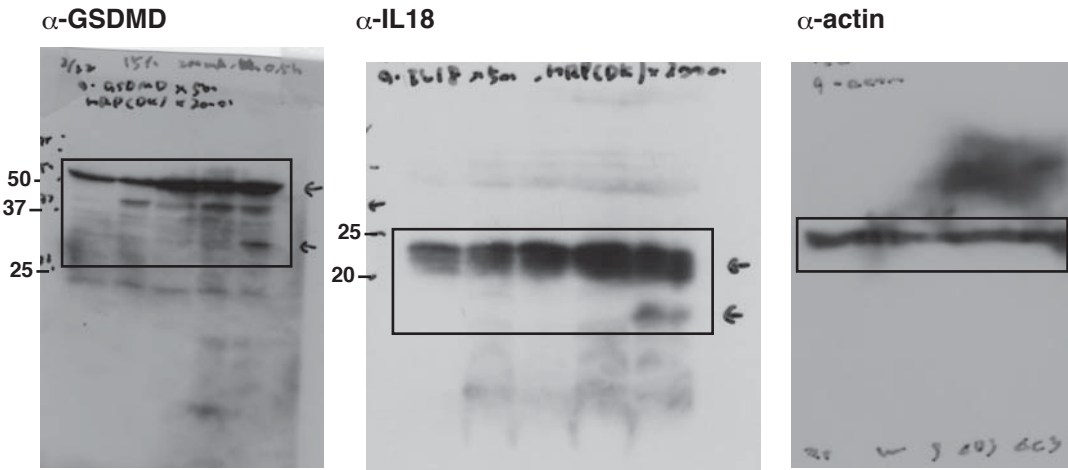

Source data for Fig. EV1

Supplement: Supplementary file 3 — Source Data for Expanded View [file EMBJ-39-e104469-s007.zip › Source data for Fig. EV1.pdf]

Fig. EV2B

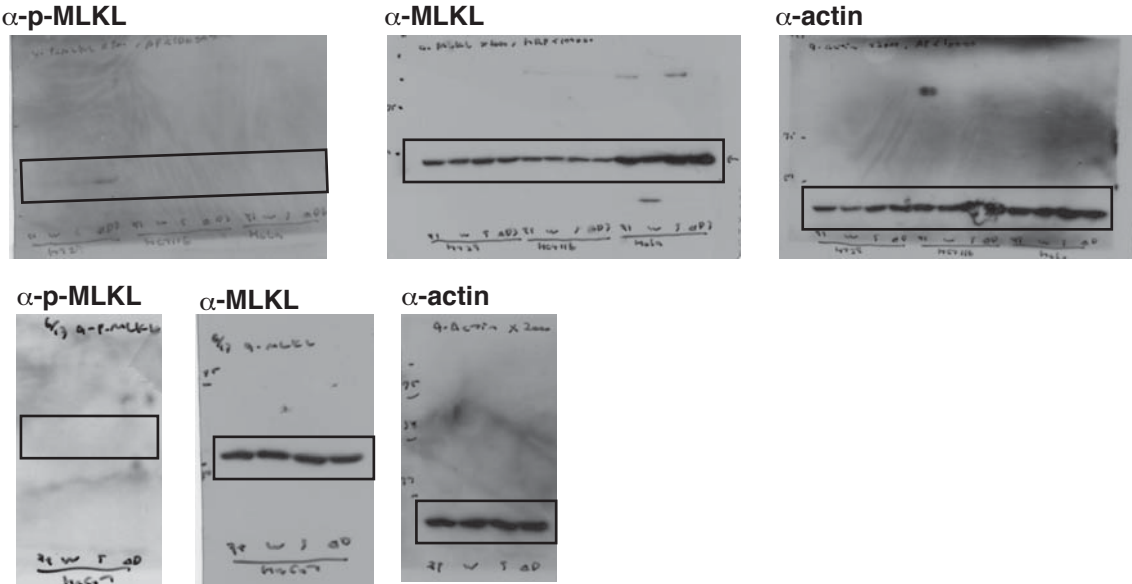

Fig. EV2C

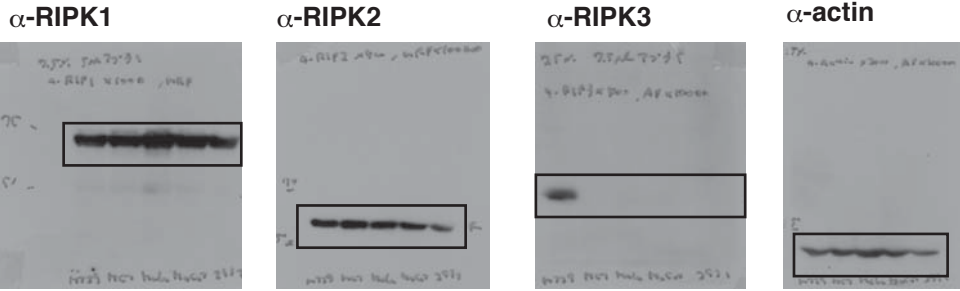

Fig. EV2D

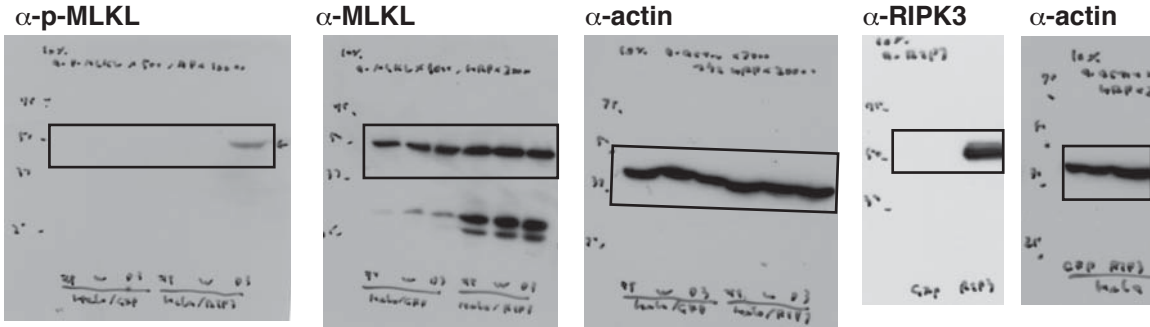

Source data for Fig. EV2

Supplement: Supplementary file 3 — Source Data for Expanded View [file EMBJ-39-e104469-s007.zip › Source data for Fig. EV2.pdf]

Fig. EV3A

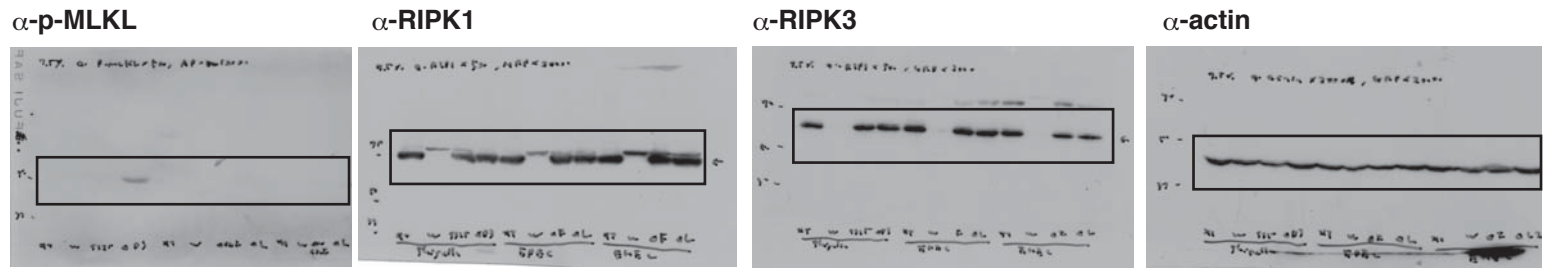

Fig. EV3C

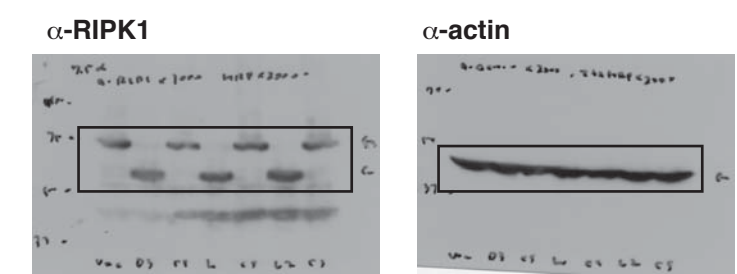

Source data for Fig. EV3

Supplement: Supplementary file 3 — Source Data for Expanded View [file EMBJ-39-e104469-s007.zip › Source data for Fig. EV3.pdf]

Fig. EV5A

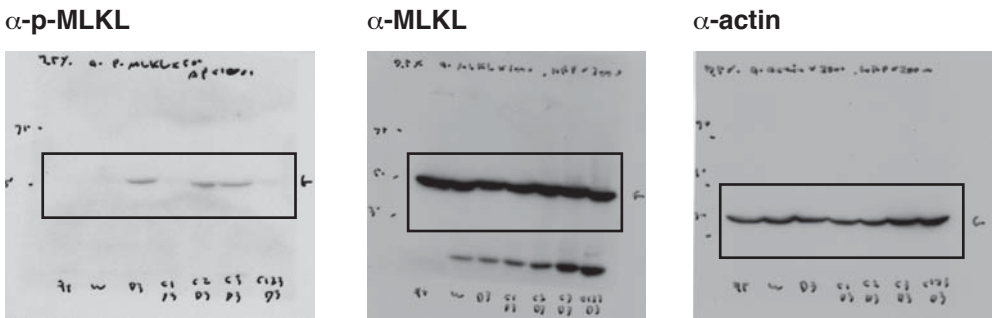

Fig. EV5B

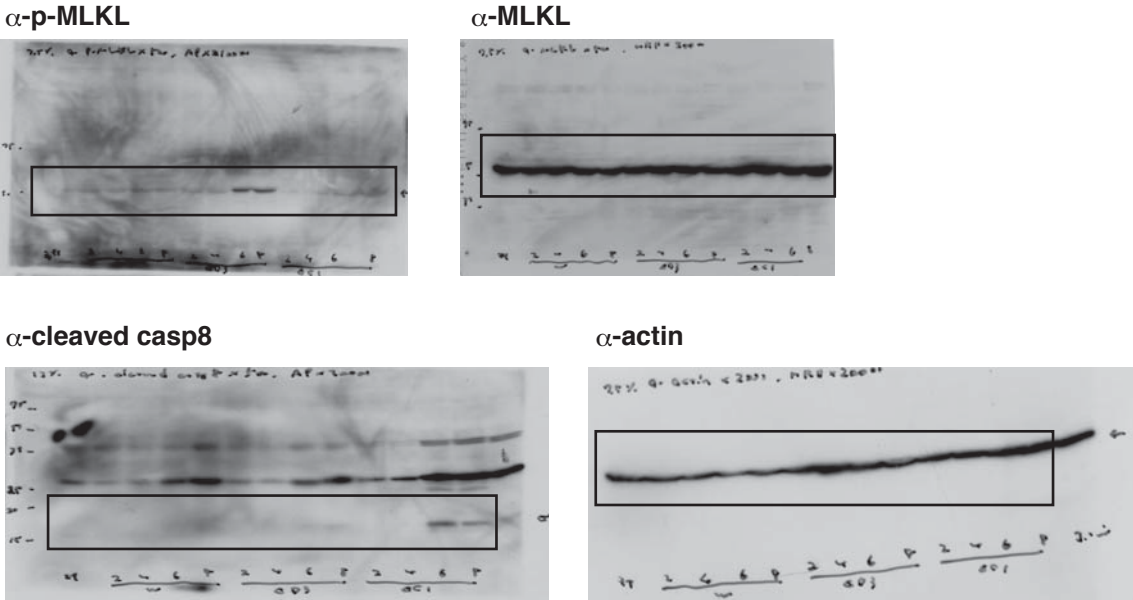

Fig. EV5C

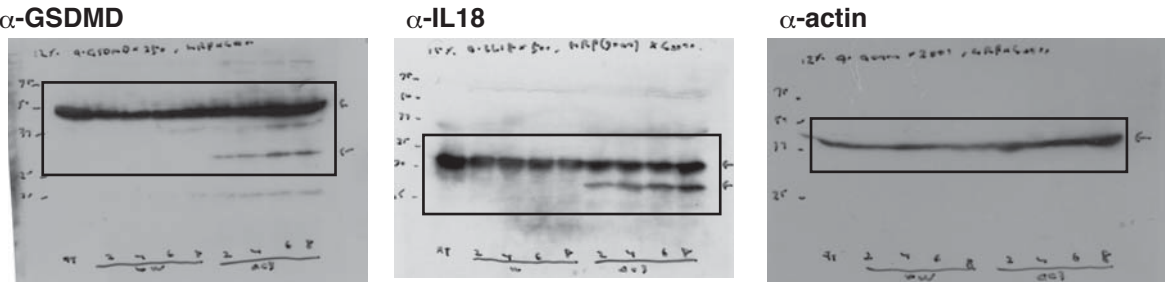

Source data for Fig. EV5

Supplement: Supplementary file 3 — Source Data for Expanded View [file EMBJ-39-e104469-s007.zip › Source data for Fig. EV5.pdf]

Fig. 2A

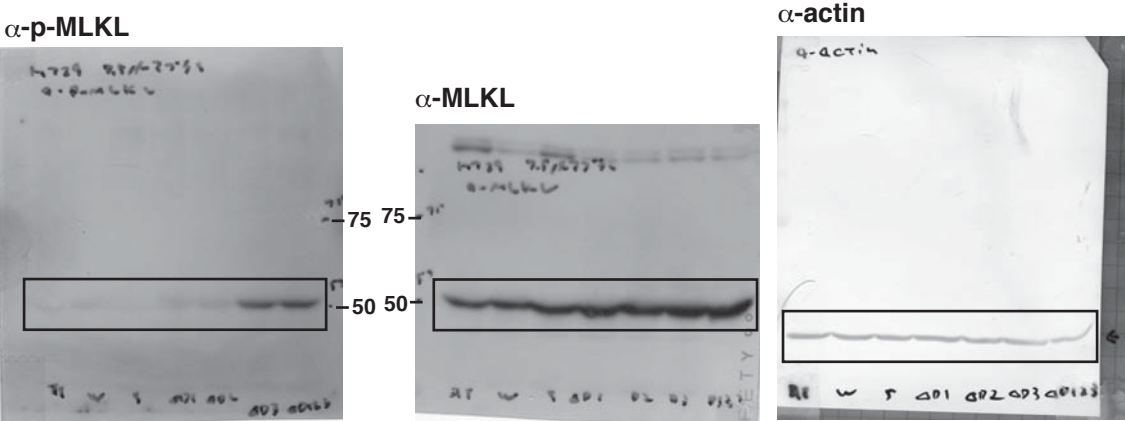

Fig. 2B

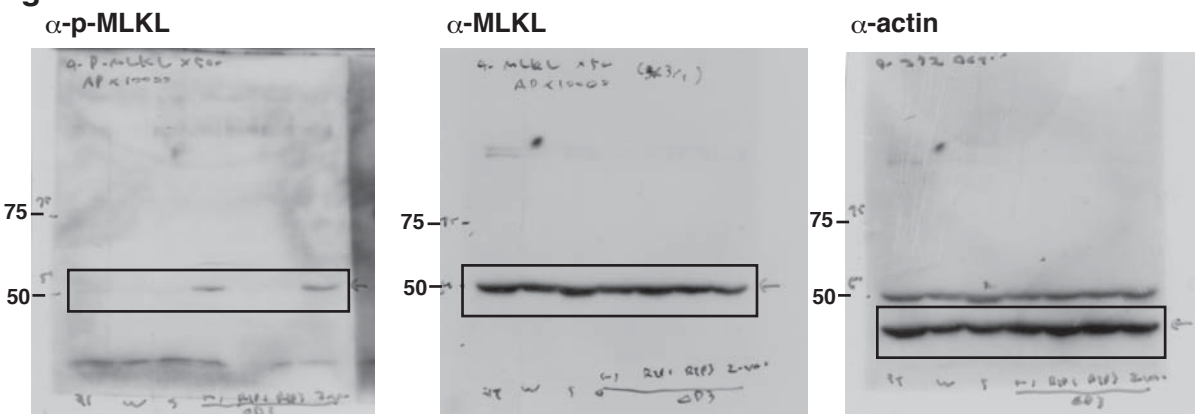

Fig. 2D

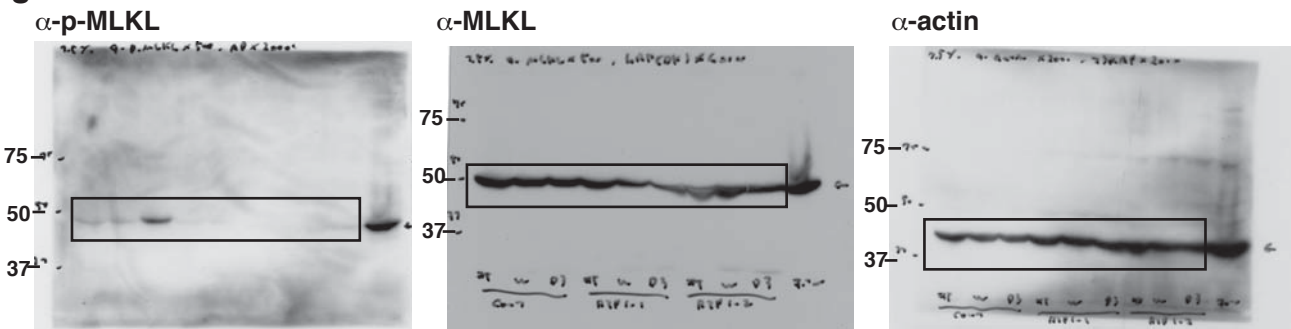

Fig. 2E

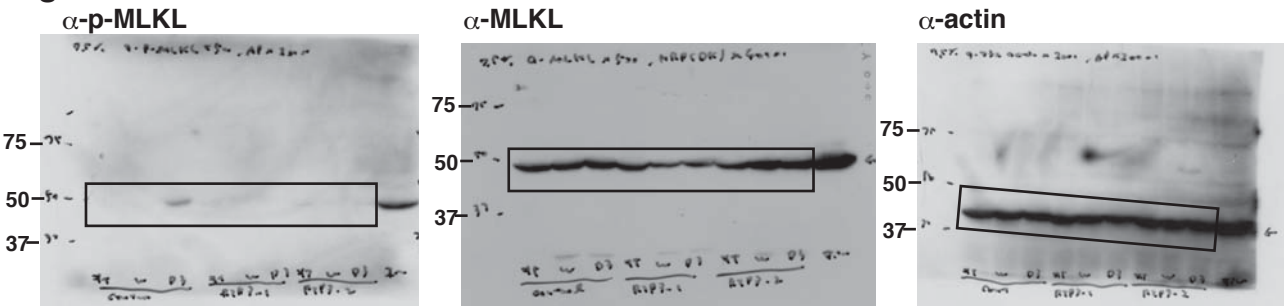

Fig. 2D (siRNA)

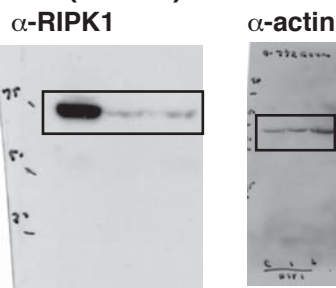

Fig. 2E (siRNA)

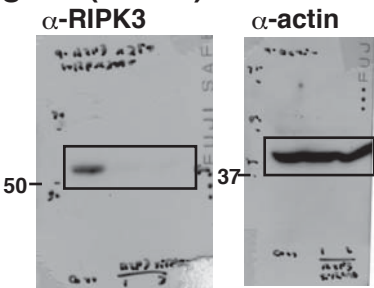

Supplement: Supplementary file 5 — Source Data for Figure 2 [file EMBJ-39-e104469-s003.pdf]

Fig. 3A

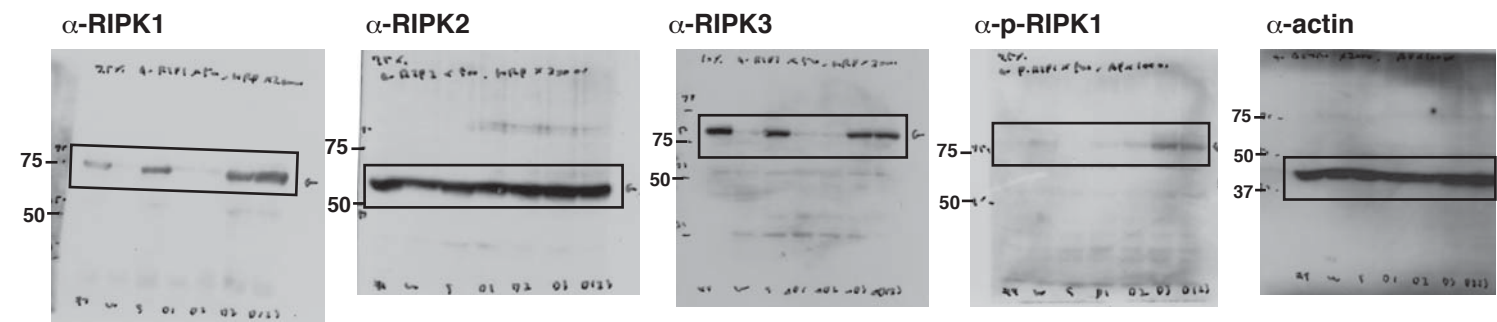

Fig. 3B

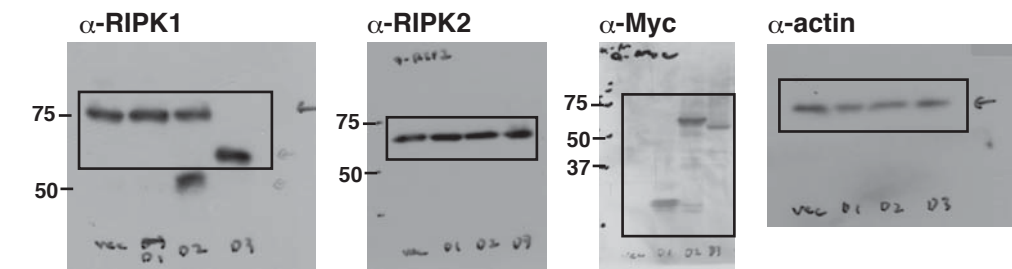

Fig. 3D

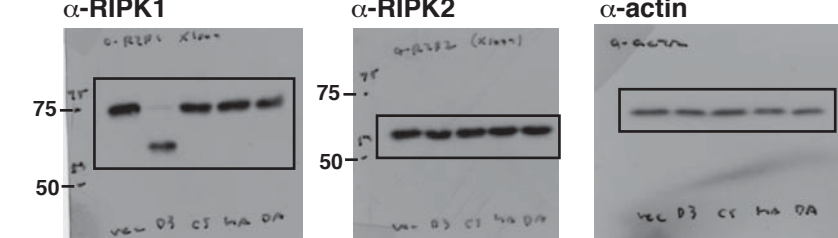

Fig. 3E

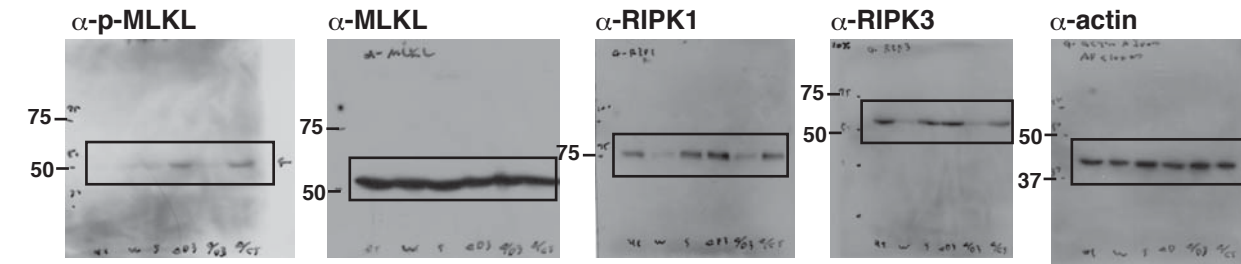

Source data for Fig. 3

Supplement: Supplementary file 6 — Source Data for Figure 3 [file EMBJ-39-e104469-s004.pdf]

**Fig. 4B**

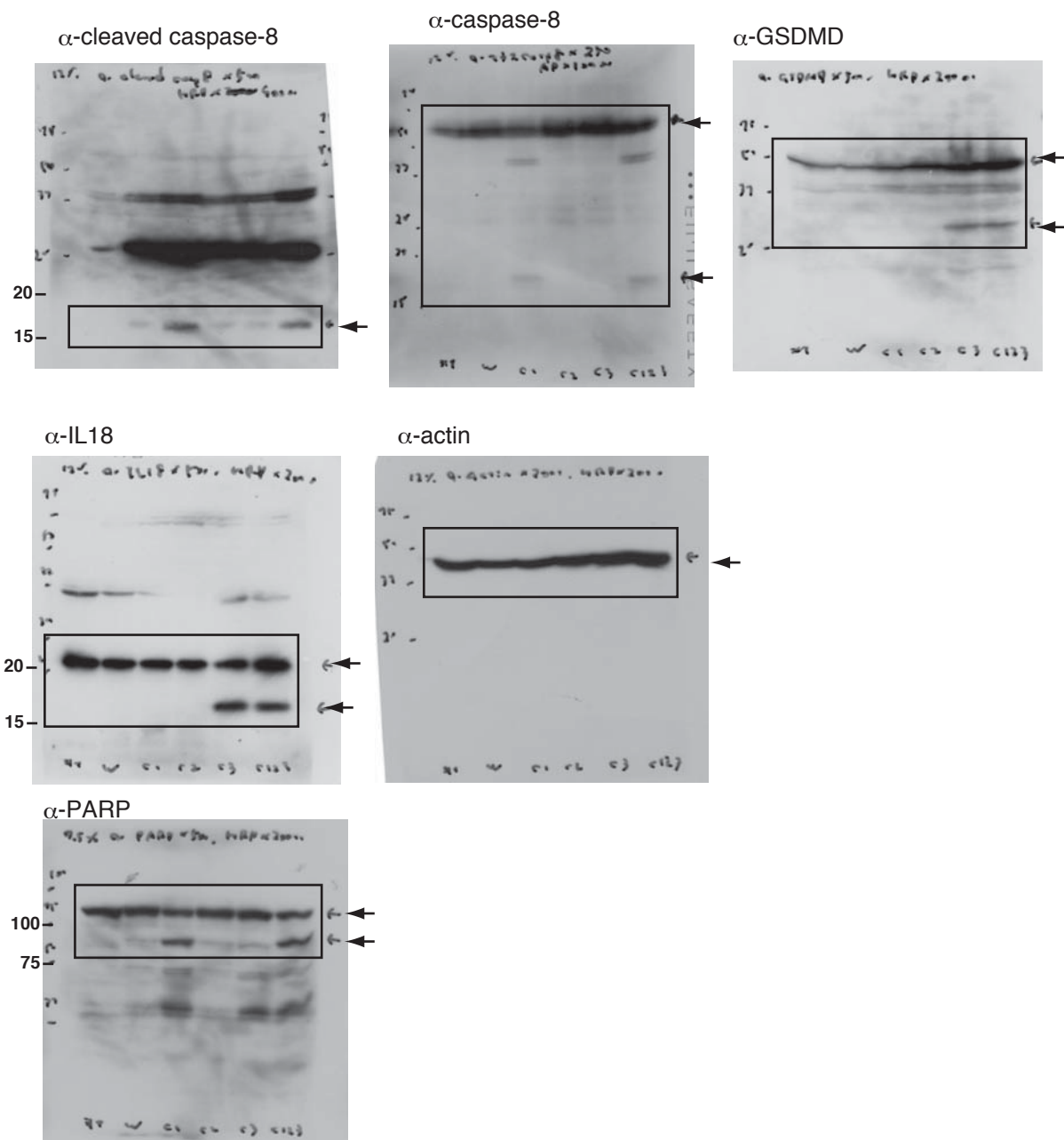

Source data for Fig. 4

Supplement: Supplementary file 7 — Source Data for Figure 4 [file EMBJ-39-e104469-s005.pdf]

Fig. 5A

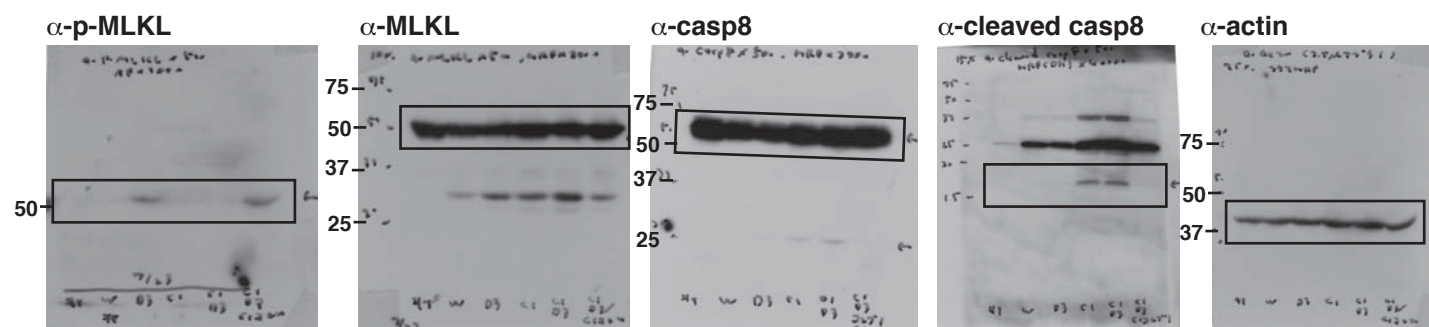

Fig. 5C

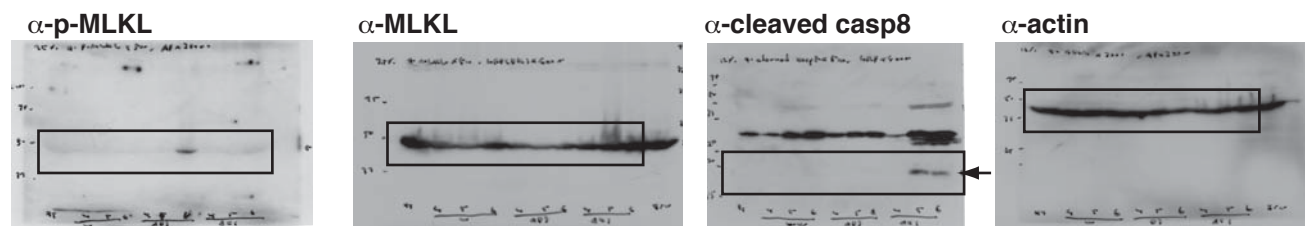

Fig. 5D

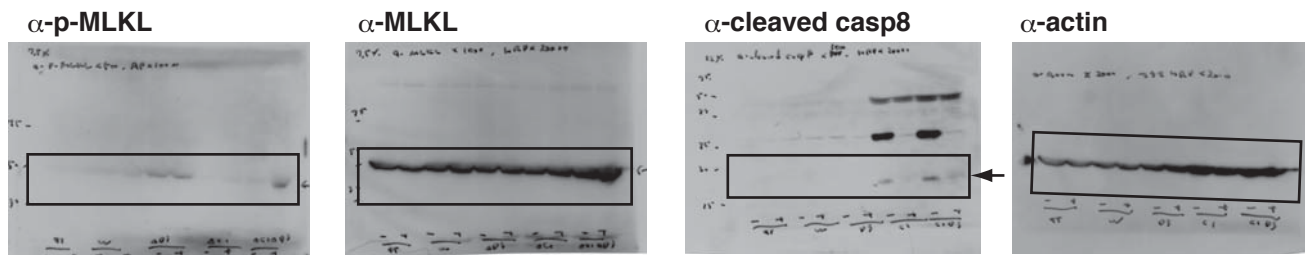

Fig. 5F

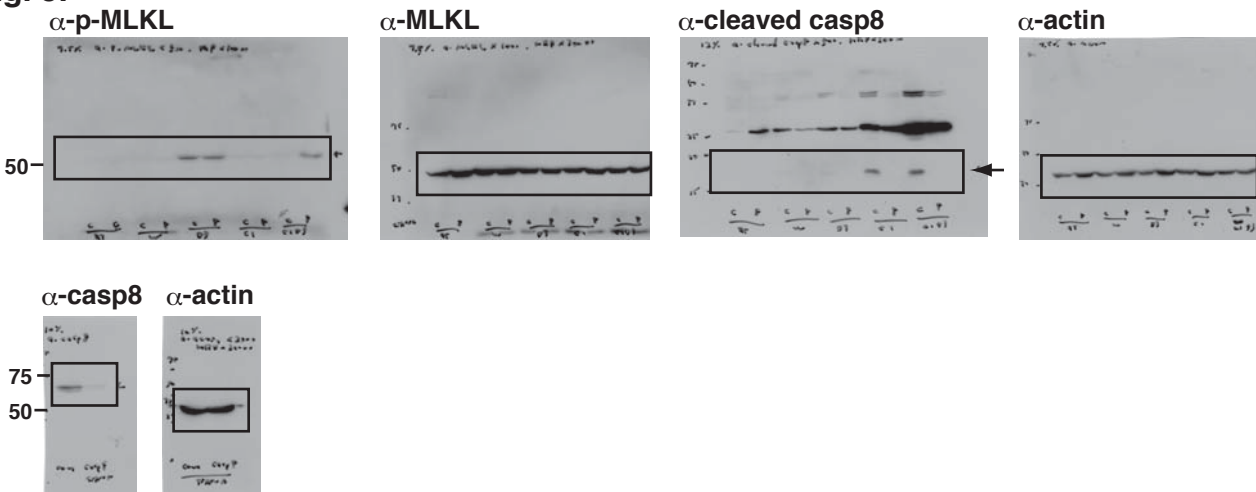

Source data for Fig. 5

Supplement: Supplementary file 8 — Source Data for Figure 5 [file EMBJ-39-e104469-s006.pdf]
